# Impact of Adalimumab Patient Support Program’s Care Coach Calls on Clinical Outcomes in Patients with Crohn’s Disease in Canada: An Observational Retrospective Cohort Study

**DOI:** 10.1093/jcag/gwy059

**Published:** 2018-10-15

**Authors:** Neeraj Narula, Brad Millson, Katia Charland, Krishna Donepudi, Tania Gaetano, Kevin McHugh, Martin G Latour, Sandra Gazel, Marie-Claude Laliberté, John K Marshall

**Affiliations:** 1 Department of Medicine, Division of Gastroenterology, Farncombe Family Digestive Health Research Institute, McMaster University, Hamilton, Ontario, Canada; 2 IQVIA, Health Access and Outcomes Division, Kirkland, Quebec, Canada; 3 AbbVie Corporation, St. Laurent, Quebec, Canada

**Keywords:** Adalimumab, Biologics, Crohn’s disease, Inflammatory bowel disease, Patient support programs

## Abstract

**Background:**

Adalimumab is an antitumour necrosis factor (TNFα) biologic therapy indicated for the treatment of Crohn’s disease (CD). Patients receiving adalimumab in Canada are eligible to enroll in the AbbVie Care™ patient support program (AC-PSP), which provides personalized services, including care coach calls (CCCs). The objective of this study was to compare the likelihood of achieving clinical remission in a cohort of CD patients treated with adalimumab who did and did not receive CCCs.

**Methods:**

A longitudinal analysis was performed using de-identified aggregate-level data collected through the AC-PSP. Patients were indexed on the date of their first injection of adalimumab between July 2010 and October 2014. The AC-PSP database included measurements of the Harvey-Bradshaw Index (HBI), a symptom-based measure of disease severity. Eligible patients had an initial HBI measurement performed between 90 days before and up to 30 days after the index date and a follow-up HBI measurement six to 18 months later. Adjusted relative risk (RR) of achieving remission (HBI ≤ 4) at the time of the follow-up was estimated comparing patients who received and did not receive CCCs.

**Results:**

There were 381 CD patients who met eligibility criteria; 224 (59%) received CCCs, and 157 (41%) did not receive CCCs. Multivariate regression analysis demonstrated that CD patients receiving CCCs had a 17% increased likelihood of achieving HBI remission when compared with patients who did not receive CCCs (RR = 1.17; 95% CI, 1.03–1.34; P = 0.0192).

**Conclusions:**

This study provides preliminary evidence that a phone call intervention, aiming to improve the overall patient experience with adalimumab treatment, may increase the likelihood of HBI remission in patients taking adalimumab to manage CD.

Crohn’s disease (CD) is a chronic and progressive disease of the colon characterized by cycles of symptomatic periods (flare-ups) and relatively symptom-free periods (remission). Crohn’s disease is one of the most disabling and costly forms of inflammatory bowel disease (IBD), and patients typically require treatment throughout their lifetime (1). Clinical guidelines recommend assessment of symptoms at the time of diagnosis and ongoing clinical assessments for patients that are on treatment, particularly biologic therapies for CD (2). The Harvey-Bradshaw Index (HBI) is a validated symptoms-based tool for assessing disease activity or response to therapy (3–5).

Adalimumab is a tumour necrosis factor-α (TNF-α) antagonist that is approved for the treatment of patients with CD in Canada. Previous studies have demonstrated that adalimumab is effective in treating patients with CD (6–8). All patients receiving treatment with adalimumab in Canada are eligible to enroll in the AbbVie Care™ Patient Support Program (AC-PSP), which was also referred to as PROGRESS when it was initially implemented. AbbVie created the AC-PSP to facilitate access to and appropriate use of adalimumab and to improve patients’ experience on adalimumab therapy. Among other services, the program provides drug reimbursement assistance, patient self-injection training, and customized support demonstrated to improve patient adherence to therapy (9). These services are aimed at improving the overall patient experience with adalimumab treatment, which in turn, could lead to better treatment outcomes. One of the innovative features unique to the AC-PSP are care coach calls (CCCs), which are calls made by trained registered nurses, known as Wellness Case Managers, to patients to provide training, education, and customized coaching, with the goal of improving patient persistence and adherence on adalimumab. In a previous Canadian study of Outcomes in Adalimumab Patients with support for adherence (COMPANION), patients with IBD who enrolled in the AC-PSP receiving CCCs demonstrated significantly greater 12-month persistence and mean medication possession ratio (MPR) to adalimumab relative to patients who did not receive CCCs (9). However, to date, no published information exists that measures the impact on clinical outcomes of CD patients, including disease remission. Thus, the overall aim of the current study was to determine if PSP services had a positive impact on HBI remission in CD patients. Specifically, the objective of this study was to compare the likelihood of achieving clinical remission in a cohort of patients with CD treated with adalimumab enrolled in the AC-PSP between the CD patients who received CCCs versus those who did not receive CCCs. We hypothesized that CD patients who received CCCs would have a higher likelihood of achieving remission based on HBI score compared with patients who did not receive CCCs.

## MATERIALS AND METHODS

### Study Design and Study Population

**Figure 1.**
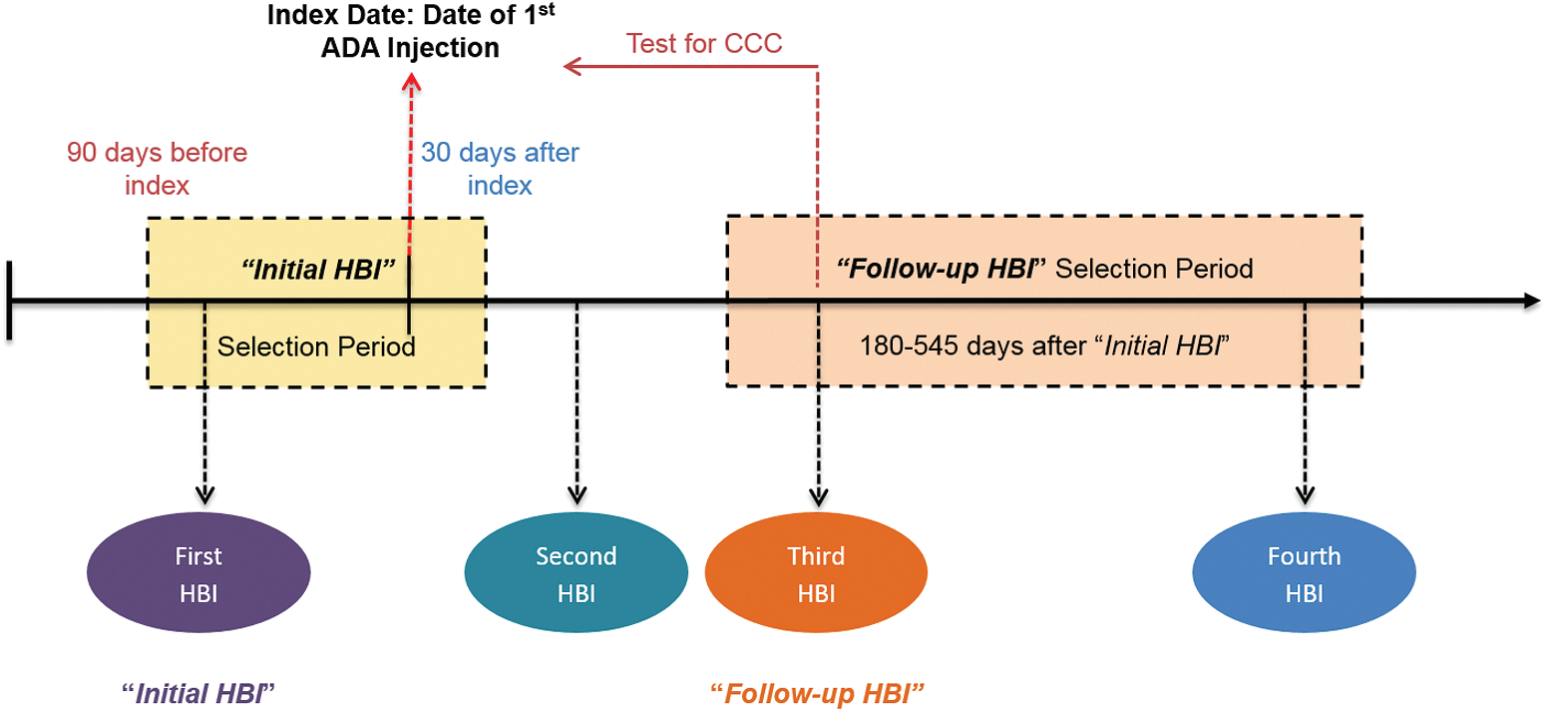
Patient selection criteria. Patients were selected for study inclusion if they had their first HBI assessment made during a period starting 90 days before and ending 30 days after the initial injection of adalimumab and had a follow-up HBI assessment made in a period beginning 180 days after and ending 545 days (six to 18 months) after the first injection date. Abbreviations: HBI, Harvey-Bradshaw Index; CCC, Coach Care Call

This was a retrospective real-world observational study. Patients were included if they were enrolled in the AC-PSP and: (1) were 18 years of age or older, (2) had a diagnosis of CD, (3) had a first injection date for adalimumab between July 1, 2010, and August 31, 2014 (index period), (4) had their first HBI assessment made during a period starting 90 days before and ending 30 days after the initial adalimumab injection, and (5) had a follow-up HBI assessment made between 180 days and 545 days (six to 18 months) after the first injection date (Figure 1). Patients with missing data including age, sex, province, claims where drug cost = 0 and where unit = 0 were excluded from the study.

To further test the robustness of the relationship between the probability of remission and receiving CCCs, an analysis was performed on a subset of patients who were confirmed to be persistent on adalimumab throughout the follow-up period. All patients from the AC-PSP cohort who met the previously mentioned criteria and who could also be linked in the IQVIA Canadian longitudinal pharmacy prescription database (LRx) were included. Only patients who were persistent during the assessment period (from adalimumab therapy start date to follow-up HBI assessment) were included in the final cohort used in the analysis. Persistence was defined as not exceeding a gap in days’ supply of adalimumab >90 days (9, 10). ‘Days’ supply’ refers to the number of days the supply of dispensed medication will last.

### Data Sources and Data Linkage

The primary analysis used retrospective data collected as part of regular operation of the AC-PSP which was further supplemented using an enhanced dataset obtained through a probabilistic matching algorithm linking the AC-PSP and IQVIA LRx database, described later on.

Data were obtained from the Canadian AC-PSP database, which contains information on patients treated with adalimumab who were enrolled in the AC-PSP. The database includes patient-level details such as patient demographics, program services rendered, patient diagnoses and treating physician information. In addition, HBI assessments were conducted by the AC-PSP Wellness Case Managers or nurses and physicians treating the patients because they correlate with disease activity and are often required by insurers for approval and ongoing reimbursement. The data used for the study were anonymized before use in the analysis.

Prescription fill patterns are not captured by the AC-PSP. Therefore, to test the objectives in a sample of patients with verified persistence on adalimumab, a subcohort of patients with confirmed persistence on adalimumab was created using the IQVIA Canadian LRx database. The LRx database captures de-identified patient-level prescription data collected from retail pharmacies across Canada and contains approximately 200 million prescriptions for more than 20 million patients, representing a capture of 75% of prescriptions nationally (11, 12). Patients using pharmacies that do not report data to IQVIA would not be captured.

A probabilistic matching algorithm was developed to link records of patients in the AC-PSP database to the LRx database (9). Probabilistic, or rule-based record linkage, finds matches using a combination of common data variables across the two datasets. All common variables in both datasets were used in the matching algorithm: sex, year of birth, prescribing physician, dispensing pharmacy, prescription fill date and prescription cost. The data linkage allowed for the study of the associations between services and interactions provided through AC-PSP and real-world patient utilization.

This linked dataset has been externally validated and used in prior published studies (9). Gerald Lebovic and Muhammad Mamdani (Institute of Health Policy, Management and Evaluation, University of Toronto, Ontario, Canada) reviewed the linked dataset and found it to be reliable. The positive predictive value of the algorithms ranged from 95.84% to 99.77%, indicating a low rate of false positives. AC-PSP patients who were linked to the IQVIA LRx database had age, sex, treatment and payer types similar to those in the overall AC-PSP population (9).

### AbbVie Care Patient Support Program Description

In order to facilitate access to reimbursement and the appropriate use of adalimumab, AbbVie implemented the AC-PSP, which provides comprehensive reimbursement assistance, injection services and patient educational and adherence support. As part of reimbursement support, clinical outcome measures such as HBI scores are recorded and provided to insurers if required for initial or ongoing approval for adalimumab. Other components of the AC-PSP include patient education, injection training, delivery and disposal of supplies, financial assistance, patient reminders and direct contact with Wellness Case Managers who deliver ongoing tailored interventions in the form of CCCs.

The CCCs were first implemented in 2013. When the CCC service was launched, it was provided to all existing and new patients (9). Patients could opt-out of receiving CCCs but did so in <1% of cases. The Wellness Case Managers provided CCCs to patients before the initiation of adalimumab and periodically over the course of treatment. These calls relied upon motivational interviewing techniques (13), with the aim of improving adherence, persistence and the overall patient experience, ultimately encouraging better health outcomes in patients being treated with adalimumab. Care coach calls were recorded and monitored for quality assurance. If there was no answer on the first CCC, three further attempts were made to reach the patient over the next five days.

### Study Outcomes

The primary outcome of interest was the likelihood of achieving remission as defined by HBI score, categorized as severe (>16), moderate (8–16), mild (5–7), and remission (≤4) (3). Improvement in HBI score was defined as moving down a category (e.g., from severe to moderate) or achieving remission. Harvey-Bradshaw Index assessments were administered independently of the CCC service described previously and did not impact the services provided to the patient. For each patient, a baseline HBI assessment was made in the period between 90 days before and up to 30 days after the date of first adalimumab injection. This period was chosen to capture the first HBI score before the full therapeutic effect of adalimumab was realized while allowing for flexibility in the first HBI assessment date. A follow-up HBI assessment was performed between six and 18 months after the baseline assessment. Only two HBI assessments were used in this analysis (i.e., the baseline and follow-up HBI score). If multiple HBI scores were collected during the follow-up period, the HBI score closest to the 12-month mark was utilized. The time period of six to 18 months allowed sufficient time for patients to respond to treatment while accommodating for variability in follow-up assessment timing seen in the real-world setting (Figure 1).

### Statistical Analysis

Comparison of differences between cohorts was performed by using the independent samples *t* test for normally distributed values and the Wilcoxon rank sum test for non-normally distributed data. The χ2 test was used for comparison of categorical data, unless cell counts were less than five, in which case the Fisher exact test was used. The likelihood of remission (i.e., HBI score ≤4) at the follow-up HBI assessment was compared in patients who received CCCs versus those who did not receive CCCs. Poisson regression with robust error variance was used to estimate the adjusted relative risk (RR) of HBI remission. Robust Poisson was used instead of logistic regression because the odds ratio from a logistic regression may largely overestimate the relative risk when the outcome is common (>10%) (14). Well over 10% of patients achieved HBI remission (Table 3). Analyses were adjusted for patient age group, sex, geographic region, prior biologic use, days lapsed between HBI assessments and baseline disease severity category (baseline HBI). Selection of covariates was based on the theoretical plausibility of the variable as a confounder of the association between receiving CCCs or not receiving CCCs and likelihood of remission (15, 16) and from previous analyses determining confounders (9). Data extraction and statistical analyses were conducted using SAS version 9.3 (SAS Institute Inc., Cary, NC).

## ETHICAL CONSIDERATIONS

Because no identifiable protected health information was extracted or accessed during the course of this study, no institutional review board review or approval was required.

Financial support for the study was provided by AbbVie. AbbVie participated in the design of the study, interpretation of data, review and approval of this publication. All authors contributed to the development of the publication and maintained control over the final content.

## RESULTS

### Baseline Patient Population Characteristics

**Figure 2.**
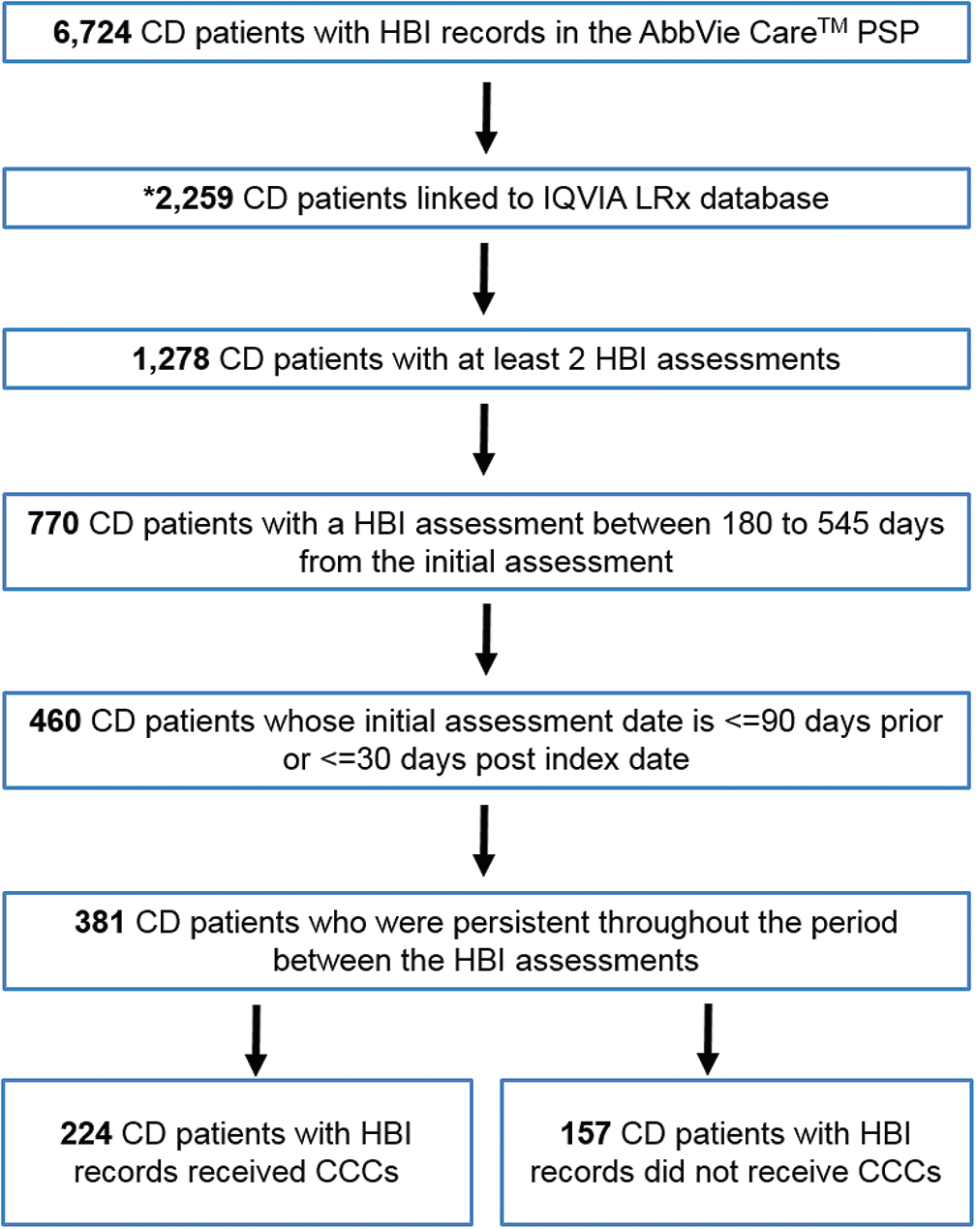
All Crohn’s disease patient selection results. Abbreviations: CD, Crohn’s disease; HBI, Harvey-Bradshaw Index; PSP, patient support program; index date, date of first adalimumab injection; CCC, care coach call. *2259 of 6724 Crohn’s disease patients could be linked between the AbbVie Care PSP and IQVIA LRx databases using a probabilistic matching algorithm described in a prior publication (9).

From the AC-PSP database, a total of 6724 patients were identified who had a CD diagnosis and ≥1 HBI assessment on file. Of these patients, 5321 were excluded because they did not meet study inclusion criteria (Figure 2). Next, an analysis on a subset of patients who were considered to be persistent in taking their medication, as they continued to pick up their adalimumab prescription at their pharmacy throughout the follow-up period, as measured using prescription records from the LRx database, was performed. The final cohort of persistent patients meeting eligibility criteria consisted of 381 patients. Of the 381 patients included in the final cohort, 224 (59%) received CCCs, and 157 (41%) did not receive CCCs.

Baseline demographic and clinical characteristics of the linked population are reported in Table 1. There were no differences in sex or days lapsed between patients who received CCCs and those who did not. Comparison of demographic factors between the cohort that received CCCs and the cohort that did not receive CCCs revealed that there were more biologic-naïve patients in the CCCs cohort (47% versus 33%; P = 0.0076). Differences in regional distribution between those patients who received CCCs and those who did not receive CCCs were also noted, with more patients receiving CCCs in Ontario in comparison with Alberta (54% versus 33%; P = 0.0005).

**Table 1.** Baseline characteristics of the linked analysis of persistent patients

Patient Characteristics	All Patients, n (%)	CCC Cohort, n (%)	No CCC Cohort, n (%)	P Value
Sex				0.7032
Male	167 (44)	100 (45)	67 (43)	
Female	214 (56)	124 (55)	90 (57)	
Age group, years				0.0168
18–29	67 (18)	35 (16)	32 (20)	
30–39	91 (24)	51 (23)	40 (26)	
40–49	60 (16)	38 (17)	22 (14)	
50–59	63 (17)	46 (21)	17 (11)	
60–69	57 (15)	25 (11)	32 (20)	
≥70 and over	43 (11)	29 (13)	14 (9)	
Region				0.0005
*West	101 (27)	74 (33)	27 (17)	
Ontario	236 (62)	121 (54)	115 (73)	
*East	44 (12)	29 (13)	15 (10)	
Biologic History				0.0076
Yes	148 (39)	74 (33)	74 (47)	
No	231 (61)	148 (66)	83 (53)	
Unknown	2 (0)	2 (1)	0 (0)	
Baseline assessment category				0.0264
Remission	22 (6)	16 (7)	6 (4)	
Mild disease	24 (6)	17 (8)	7 (4)	
Moderate disease	256 (67)	155 (69)	101 (64)	
Severe disease	79 (21)	36 (16)	43 (27)	
Remission				0.0035
Yes	280 (73)	177 (79)	103 (66)	
No	101 (27)	47 (21)	54 (34)	
Gap between baseline and follow-up HBI assessment, median days (IQR)	402.0 (117.0)	401.5 (117.5)	404.0 (116.0)	0.2785

Abbreviations: CCC, care coach call; HBI, Harvey-Bradshaw Index; IQR, interquartile range

*West consisted of patients from Alberta and East consisted of patients from PE, NL, NB ad NS

Values are numbers (percentages) unless stated otherwise. Comparison of differences between groups was performed by using the independent samples *t* test for normally distributed variables and the Wilcoxon rank sum test for non-normally distributed variables. The χ2 test was used for comparison of categorical data, unless cell counts were less than five, in which case the Fisher exact test was used

### Follow-up Patient Population Characteristics

In this linked patient cohort, 344 patients (90.3%) experienced an improvement in disease severity six to 18 months after HBI index assessment (Table 2). Twenty-two (92%), 198 (77%), and 43 (54%) patients with mild, moderate, and severe disease activity at baseline were in remission at the follow-up HBI assessment, respectively. Furthermore, 17 patients (77%) in remission at baseline remained in remission at follow-up.

**Table 2.** Change in assessment category of the linked analysis of persistent patients

Assessment Category	Total		CCC Cohort		No CCC Cohort	
	n	%	n	%	n	%
Change in Assessment Category						
Remission No Change	17	77%	13	81%	4	67%
Mild Disease Improved to Remission	22	92%	16	94%	6	86%
Moderate Disease Improved to Remission	198	77%	126	81%	72	71%
Severe Disease Improved to Remission	43	54%	22	61%	21	49%
Overall Change in Assessment Category						
Increase in Severity	6	2%	3	1%	3	2%
No Change	31	8%	23	10%	8	5%
Improvement/Decrease in Severity	344	90%	198	88%	146	93%
**Total**	**381**	**100%**	**224**	**100%**	**157**	**100%**

Abbreviation: CCC, care coach call

### Multivariable Regression Analysis

The proportion of patients in remission was 79% for patients receiving CCCs versus 66% for patients who did not receive CCCs, indicating a 20% increased likelihood of remission in patients with CCC compared with those without CCC before controlling for confounders. After adjustment for baseline severity, age, gender, geographic region, biologic-naïve status and the number of days between assessments, receiving CCCs was associated with a 17% increased probability of achieving remission (RR 1.17; 95% CI, 1.03–1.34; P = 0.0192 (Table 3)).

**Table 3.** Results of multivariable regression analysis of the linked analysis of persistent patients

Patient Characteristics	All Patients, n (%)	CCC Cohort, n (%)	No CCC Cohort, n (%)	Relative Risk	Lower 95% CI	Upper 95% CI	*P* Value
Sex							
Male*	167 (44)	100 (45)	67 (43)	1			
Female	214 (56)	124 (55)	90 (57)	1.081	0.9502	1.2296	0.2361
Age group, years							
18–29*	67 (18)	35 (16)	32 (20)	1			
30–39	91 (24)	51 (23)	40 (26)	0.8551	0.709	1.0312	0.1015
40–49	60 (16)	38 (17)	22 (14)	0.8773	0.7156	1.0754	0.2078
50–59	63 (17)	46 (21)	17 (11)	0.9192	0.7573	1.1158	0.3945
60–69	57 (15)	25 (11)	32 (20)	0.8803	0.6953	1.1146	0.29
≥70 and over	43 (11)	29 (13)	14 (9)	0.947	0.7738	1.159	0.5978
Region							
West	101 (27)	74 (33)	27 (17)	0.8879	0.7761	1.0293	0.1149
Ontario*	236 (62)	121 (54)	115 (73)	1			
East	44 (12)	29 (13)	15 (10)	1.0561	0.8756	1.2737	0.5677
Biologic History							
Yes	148 (39)	74 (33)	74 (47)	0.9371	0.8251	1.0642	0.3173
No*	231 (61)	148 (66)	83 (53)	1			
Baseline assessment category							
Remission	22 (6)	16 (7)	6 (4)	1.4283	1.052	1.9394	0.0223
Mild disease	24 (6)	17 (8)	7 (4)	1.7153	1.3326	2.2078	<0.0001
Moderate disease	256 (67)	155 (69)	101 (64)	1.4317	1.1565	1.7723	0.001
Severe disease*	79 (21)	36 (16)	43 (27)	1			
Remission							
Yes	280 (73)	177 (79)	103 (66)	1.1724	1.0263	1.3393	0.0192
No	101 (27)	47 (21)	54 (34)	1			
Gap between baseline and follow-up HBI assessment, median days (IQR)	402.0 (117.0)	401.5 (117.5)	404.0 (116.0)	1.0006	0.9999	1.0013	0.0761

Abbreviations: CCC, care coach call; CI, confidence interval; HBI, Harvey-Bradshaw Index; IQR, interquartile range.

*Indicates reference category within a particular variable. Values are numbers (percentages) unless stated otherwise.

## DISCUSSION

This retrospective real-world analysis linked AC-PSP data (i.e., CCCs and HBI assessments) to a longitudinal pharmacy prescription database to identify patients who were diagnosed with CD, enrolled in the AC-PSP program and persistently treated with adalimumab. This study found that patients with CD who received tailored coaching consultations (i.e., CCCs) with a Wellness Care Manager were significantly more likely to achieve HBI remission than those patients who did not receive this coaching service. This is the first time real-world evidence has been used to evaluate the impact of PSP services in Canada in the setting of adalimumab therapy.

Panaccione et al. previously evaluated open-label adalimumab therapy (16) in Canadian patients with moderate to severe CD who were either naïve to or previously exposed to anti-TNFα therapy and found that adalimumab therapy induced and sustained steroid-free remission in both infliximab-experienced and anti-TNFα-naïve patients with moderate to severe CD. Specifically, clinical remission (HBI score of ≤4) at week 24 was achieved by 53% of patients who were anti-TNF-naïve and 36% of patients who were infliximab-experienced (P < 0.01; P < 0.001 for both groups for all visits versus baseline) (16). The current study demonstrated higher rates of clinical remission. Overall, at the time of the follow-up HBI assessment, 86% of patients experienced an improvement in disease severity between six and 18 months after the baseline HBI assessment. According to their baseline disease severity category, 83%, 72% and 52% of patients with mild, moderate and severe disease, respectively, experienced remission.

Enrollment in a PSP has also been associated with increased adherence (17, 18). Results from a previous analysis of the Canadian AC-PSP indicated that 43% of patients in the AC-PSP had a ≥80% medication possession ratio (MPR) over 12 months (9). In this study, Marshall et al. reported that patients with CD and ulcerative colitis who were enrolled in the AC-PSP and who received CCCs were 69% less likely to stop treatment (hazard ratio, 0.306; P < 0.0001), and were 37% more likely to be highly adherent (≥80% MPR) than those who did not receive a CCC (odds ratio, 1.365; P = 0.0004) (9). The observed improvement in HBI outcomes for patients receiving CCCs may be due to improved persistence and adherence associated with receiving CCCs.

There is also general agreement among professionals involved in managing IBD that nurses, as part of a multidisciplinary team, play an important role with respect to providing additional patient support (19, 20). Within PSPs, healthcare practitioners, including nurses, represent a key point of access for patients for education and information and are also a means for patients to share and discuss the impact of disease on everyday life and specific symptom difficulties (19, 20). Several studies have reported improvements in patient outcomes when a dedicated IBD nurse was involved in patient care, including fewer hospital admissions (21–24), reduced length of hospital stay (23, 24) and temporary improvements in health-related quality of life (25). This is the first study demonstrating the impact of a multidisciplinary team PSP service provided by a Wellness Case Manager on clinical outcomes in patients with CD.

Our study was conducted on a robust population-based sample of 381 patients who were representative of all regions of Canada. However, the analysis is subject to several limitations. The first limitation of this study, consistent with its retrospective observational design, is that patients were not randomly assigned to receive CCCs. Due to a lack of randomization in secondary analysis of real-world evidence, there will always be confounding in these types of studies (26). To account for this, multivariate analyses were undertaken to account for confounding factors. In addition, patients enrolled before 2013 (when CCCs were introduced) were included in this analysis as controls. Nonetheless, the retrospective nature of this study is subject to inherent bias that cannot be completely eliminated by study design or statistical techniques. Caution must be applied when extrapolating this study’s findings to the larger Crohn’s disease population. Second, the probabilistic matching algorithm is subject to false links between the LRx and AC-PSP databases. However, an external review of the approach found the positive predictive value of the algorithms ranged from 95.84% to 99.77%, indicating a low rate of false positives (9). Third, although the HBI is a validated tool, it can only provide an indirect measure of disease activity, and its actual incorporation into a clinician’s day to day practice may be prone to variability and to practitioner bias, even though insurers require HBI assessments for reimbursement.

This retrospective observational study provides preliminary evidence that a phone call intervention aiming to improve the overall patient experience with adalimumab treatment may increase the likelihood of remission in patients taking adalimumab to manage CD. The intervention (CCCs) and outcomes (remission) were derived from secondary data analysis of the AC-PSP dataset. Remission was defined as having an HBI ≤4. Future research, employing a prospective design and evaluating remission by additional modalities (e.g., endoscopy), is required to validate this study’s findings and verify if they can be extrapolated to the general population of Crohn’s disease patients.
